# Impact of Polymers on Magnesium-Based Hydrogen Storage Systems

**DOI:** 10.3390/polym14132608

**Published:** 2022-06-27

**Authors:** Sadhasivam Thangarasu, Tae Hwan Oh

**Affiliations:** School of Chemical Engineering, Yeungnam University, Gyeongsan 38541, Korea

**Keywords:** hydrogen energy, magnesium hydride, MgH_2_, Mg nanoparticles, metal hydride, intermetallic hydride, gas selective polymer, poly(methyl methacrylate), hydrogen uptake

## Abstract

In the present scenario, much importance has been provided to hydrogen energy systems (HES) in the energy sector because of their clean and green behavior during utilization. The developments of novel techniques and materials have focused on overcoming the practical difficulties in the HES (production, storage and utilization). Comparatively, considerable attention needs to be provided in the hydrogen storage systems (HSS) because of physical-based storage (compressed gas, cold/cryo compressed and liquid) issues such as low gravimetric/volumetric density, storage conditions/parameters and safety. In material-based HSS, a high amount of hydrogen can be effectively stored in materials via physical or chemical bonds. In different hydride materials, Mg-based hydrides (Mg–H) showed considerable benefits such as low density, hydrogen uptake and reversibility. However, the inferior sorption kinetics and severe oxidation/contamination at exposure to air limit its benefits. There are numerous kinds of efforts, like the inclusion of catalysts that have been made for Mg–H to alter the thermodynamic-related issues. Still, those efforts do not overcome the oxidation/contamination-related issues. The developments of Mg–H encapsulated by gas-selective polymers can effectively and positively influence hydrogen sorption kinetics and prevent the Mg–H from contaminating (air and moisture). In this review, the impact of different polymers (carboxymethyl cellulose, polystyrene, polyimide, polypyrrole, polyvinylpyrrolidone, polyvinylidene fluoride, polymethylpentene, and poly(methyl methacrylate)) with Mg–H systems has been systematically reviewed. In polymer-encapsulated Mg–H, the polymers act as a barrier for the reaction between Mg–H and O_2_/H_2_O, selectively allowing the H_2_ gas and preventing the aggregation of hydride nanoparticles. Thus, the H_2_ uptake amount and sorption kinetics improved considerably in Mg–H.

## 1. Introduction

The hydrogen economy showed considerable benefits in the industrial system at the present moment worldwide, which has realized the importance of the combination of hydrogen and electricity together [1,2,3]. Hydrogen energy is considered an efficient energy carrier for present and future energy needs. Hydrogen energy has been considered a primary alternative to replace fossil fuels, where it can be utilized as a fuel for automobiles and provide heat/electricity for industries and households [4,5,6]. The researchers recognize that hydrogen energy possesses numerous advantages, such as

- Its abundance and lightweight;
- Clean and green energy system (eco-friendly and clean source of energy);
- The by-product is only water without any greenhouse gas emissions or any harmful emissions during the energy conversion;
- Higher energy conversion rate (the energy of H_2_ is approximately 3 times higher than petroleum fuels;
- Possibilities to derive from various sources and different methods;
- Reliable and sustainable energy system.

The key aspects of hydrogen energy are hydrogen production, storage and distribution [7,8]. For hydrogen production, numerous kinds of technologies are executed to produce the hydrogen in feasible ways [9,10,11,12]. Hydrogen production from renewable energy sources can be considered an efficient technique (such as electrolysis/photocatalytic of water splitting) because of its high purity and hydrogen production without any harmful emissions [13,14,15,16,17,18,19]. The produced hydrogen can be stored in the gaseous, liquid and solid states [7,20]. The electricity can be derived from the stored hydrogen using fuel cells without toxic emissions [21,22,23,24]. During fuel cell operation, the electricity is generated in an anode electrode. The electrons traveled to the cathode via an external circuit, and protons are transported via membranes. The overall electrochemical reaction was completed on the cathode via an oxygen reduction reaction [25,26,27]. Apart from the numerous developments and advantages in hydrogen production, storage and distribution, inevitable drawbacks are associated with each system. For instance, the following problems are associated with the electrochemical systems of water splitting and fuel cells such as (i) high cost and rarity of electrocatalyst materials, (ii) sintering, migration and aggregation of electrocatalysts, (iii) high production cost of the membrane, and (iv) corrosion of bipolar plates [28]. Numerous efforts have been implemented to overcome the issues in hydrogen production and the utilization system [29,30,31,32,33,34,35]. Apart from this, serious concerns have been provided to the hydrogen storage sectors because certain demerits need to be overcome, such as lower volumetric/gravimetric density of hydrogen, thermodynamic-related issues in material-based hydrides, sorption kinetics, contamination during exposure to air and mainly storage capacity/conditions [36,37,38,39]. Numerous efforts have been proposed to overcome these issues in hydrogen storage materials (e.g., magnesium (Mg)-based hydrides). From this viewpoint, polymeric materials have been effectively considered for metal hydrides to overcome contamination-related issues and thermodynamic barriers. Moreover, the polymers are widely used in the rechargeable Mg and M-H batteries as polymer electrolytes and electrode materials [40,41,42,43,44,45]. The polymer materials are used as electrode materials in rechargeable Mg batteries because of their unique properties such as being cost-effective, lightweight, recyclable, flexible structural tunability, enhancing the reaction kinetics due to efficient interaction with active species and facilitating reversible electrochemical interactions by weak intermolecular forces [40,41]. Likewise, polymers are commonly used in energy and environmental devices as active materials, electrolytes, binders for electrodes and separators/membranes [46,47,48,49,50]. Here, we provide a comprehensive review of the fundamentals of hydrogen storage systems and importance of the magnesium (Mg) and magnesium hydride (MgH_2_). In this review, the focuses provide as follows (i) limitations in Mg/MgH_2_ systems and possible options to overcome its limitations, (ii) a list of polymer materials used with hydrides, specifically Mg/MgH_2_ and (iii) the impact of polymer materials on altering the properties and performances of Mg/MgH_2_. Finally, several approaches and combinations of polymer and Mg/MgH_2_ have been reviewed. The role and importance of polymer materials in every consecutive section (carboxymethyl cellulose, polystyrene, polyimide, polypyrrole, polyvinylpyrrolidone, polyvinylidene fluoride, polymethylpentene, and poly(methyl methacrylate)) with hydrogen storage materials have been described. This review can provide a better direction in this field to develop an advanced in polymer-hydride composite.

## 2. Significance of Magnesium Hydride and Benefits of Polymeric Materials for Hydrides

**Figure 1:**
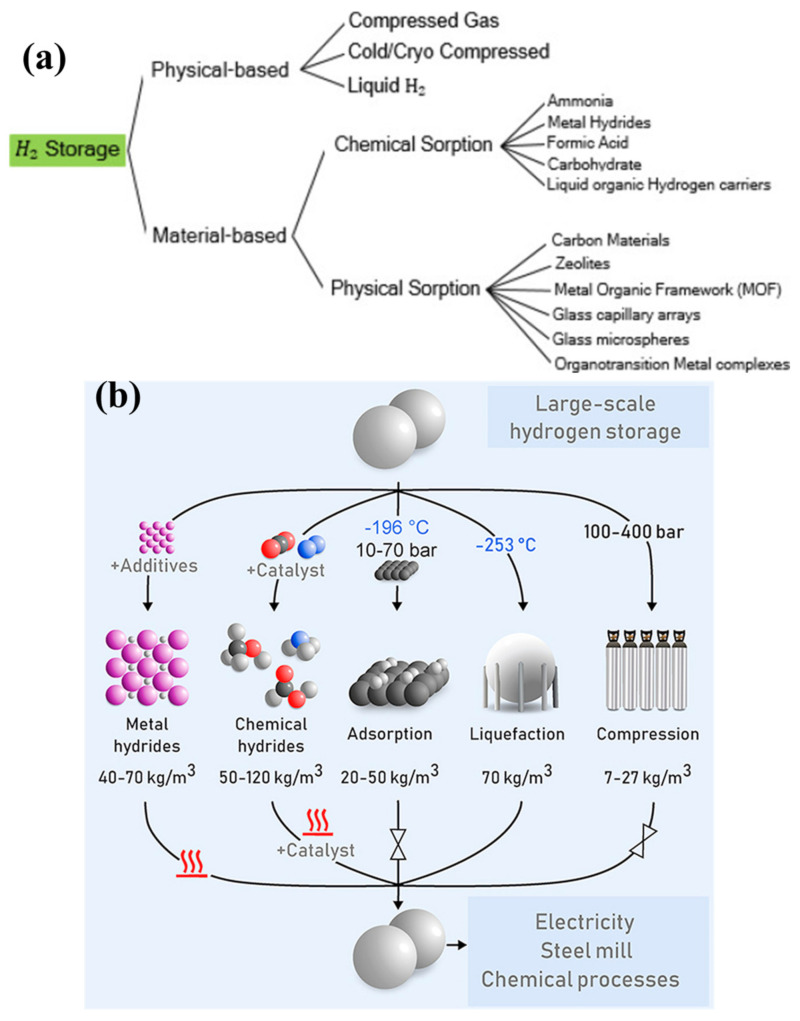
(**a**) Classification of hydrogen storage methods. Reprinted with permission from Ref. [7]. Copyright © 2019 Hydrogen Energy Publications LLC. Published by Elsevier Ltd. (License Number: 5304790559586). (**b**) Hydrogen storage amounts in different methods [8].

**Figure 2:**
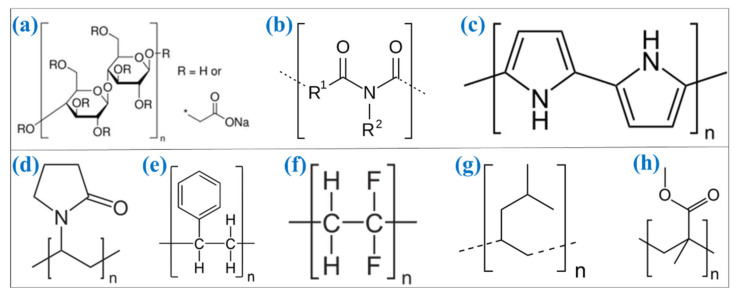
Chemical structure of polymers used with Mg-based hydrides: (**a**) carboxymethyl cellulose, (**b**) polyimide, (**c**) polypyrrole, (**d**) polyvinylpyrrolidone, (**e**) polystyrene, (**f**) polyvinylidene fluoride, (**g**) polymethylpentene and (**h**) poly(methyl methacrylate).

Hydrogen can be stored in two broad categories: physical-based and material-based hydrogen storage systems, as shown in Figure 1 [7]. In physical-based hydrogen storage systems, hydrogen can be stored in three categories: compressed gas, cold/cryo compressed and liquid hydrogen [7,51,52]. In material-based hydrogen storage systems, the hydrogen stored in various categories such as adsorbent (e.g., metal–organic framework) [53,54,55], liquid organic [56,57,58] (e.g., BN-methyl cyclopentane), interstitial hydride (e.g., LaNi_5_H_6_) [59,60], elemental hydride (e.g., MgH_2_) [61,62,63,64], complex hydride (e.g., NaAlH_4_) [65,66,67] and chemical hydrogen (NH_3_BH_3_) [7,51,68,69,70] (Hydrogen Storage, Hydrogen and Fuel Cell Technologies Office, US-DOE—https://www.energy.gov/eere/fuelcells/hydrogen-storage, accessed on 2 April 2022). Pressurized hydrogen gas is commonly used in various applications in the present scenario. However, the challenges are associated with lower volumetric density (0.08988 g/L) of hydrogen [38,71], where high-pressure techniques are needed to store more hydrogen in the gaseous state [20]. It remains not easier to utilize in transportation and household applications [72,73]. Another form of hydrogen storage in a liquid state can store more hydrogen [74,75]. However, it has to be maintained in the cryogenic conditions, and probably, additional energy is required to maintain this condition [76]. The safe and efficient technique is to store the hydrogen in the solid state of material-based hydrogen storage systems [77,78]. In material-based hydrogen storage systems, the hydrogen can be stored in two forms: (i) physical adsorption on the material and (ii) stored through chemical bonds [79,80]. Among the different hydrogen storage materials, magnesium (Mg) has been considered one of the excellent materials for hydrogen storage material because of its different forms in hydrides [81,82,83,84]. Mg can be formed as intermetallic, elemental and complex hydrides, such as magnesium hydride (MgH_2_) [85,86], Mg_2_NiH_x_ [87,88], Mg(AlH_4_)_2_ [89,90] and Mg(BH_4_)_2_ [91,92,93,94]. The Mg/MgH_2_ has been considered one of the efficient hydride materials because of its numerous advantages, such as high gravimetric hydrogen capacity (7.6 wt %), high volumetric hydrogen capacity (110 g hydrogen/liter), excellent reversibility at nanoscale and stability during cycle performances [62,95,96,97]. The gravimetric and volumetric hydrogen capacity of Mg/MgH_2_ meets the US-DOE technical targets as represented in Table 1 [5] (DOE Technical Targets for Onboard Hydrogen Storage for Light-Duty Vehicles—https://www.energy.gov/eere/fuelcells/doe-technical-targets-onboard-hydrogen-storage-light-duty-vehicles, accessed on 28 April 2022). Apart from the numerous advantages, it possesses certain demerits such as high desorption temperature, slower sorption kinetics, limiting the reversibility in bulk state, and oxidization in ambient conditions [98,99,100]. Numerous kinds of efforts have been made to improve the sorption kinetics and alter the thermodynamic-related issues by including catalyst materials [101,102,103,104], changing the dimensional [91,105,106,107] and confining in porous structures [108,109,110]. However, these kinds of efforts cannot effectively prevent the Mg-based hydrides from being contaminated when the hydrides are exposed to air. Recently, the combination of hydride materials with polymer has shown considerable benefits in preventing the hydride materials from the air and moisture contaminants [39]. Additionally, the polymers can effectively store the hydrogen in certain conditions [111,112,113]. The polymer-covered hydride materials provide confinement and a core–shell structure, making them one of the efficient options for overcoming the contamination-related issues in the hydride materials. The polymers containing gas-selective properties can effectively be considered an excellent candidate for controlling the contaminants’ interaction with hydrides. In this case, the polymers selectively allow the hydrogen gas and act as a barrier for other gases and protic species [39,114]. Moreover, the polymeric material provides numerous benefits for hydride materials, such as preventing the aggregation of hydride material during the hydrogen sorption cycle, stabilizing the hydride material’s dimension and improving the hydrogen sorption behavior. Based on these advantages, many polymers have been studied for different hydrogen storage materials [115,116,117,118,119,120,121,122,123,124]. For magnesium hydride systems, very few polymers (as represented in the chemical structure of polymers in Figure 2), namely carboxymethyl cellulose, polystyrene, polyimide, polypyrrole, polyvinylpyrrolidone, polyvinylidene fluoride, polymethylpentene, and poly(methyl methacrylate) have been effectively considered to develop a new kind of magnesium hydride–polymer composite for prohibiting the contamination in magnesium hydride systems.

## 3. Carboxymethyl Cellulose

**Figure 3:**
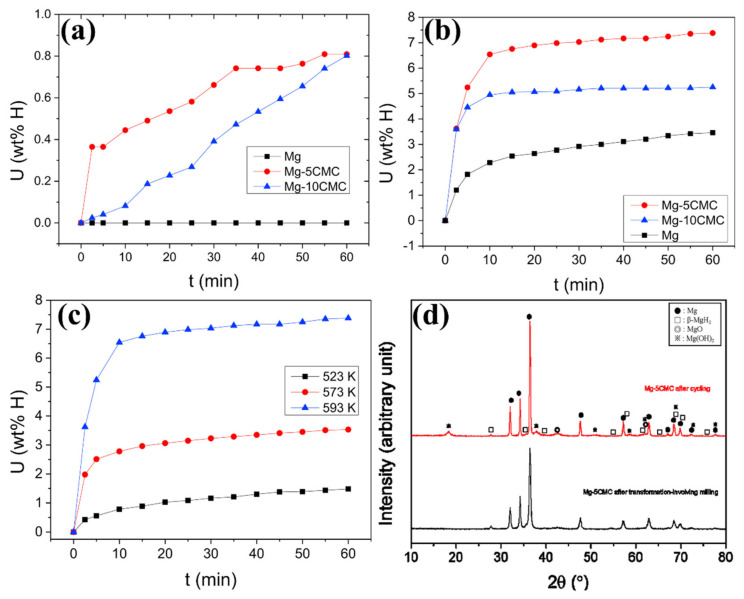
Hydrogen absorption of Mg, Mg-5CMC and Mg-10CMC at (**a**) 523K and (**b**) 593K. (**c**) Hydrogen absorption of Mg-5CMC at different temperatures. (**d**) XRD of Mg-5CMC before and after cycle performances. Reprinted with permission from Ref. [129]. Copyright ©2018 Hydrogen Energy Publications LLC. Published by Elsevier Ltd. (License Number: 5305030041872).

The semi-synthetic polymers have been utilized in various applications because of their functional properties. The semi-synthetic cellulose polymer can be derived from natural cellulose such as cotton and wood with further modifications, where the cellulose derivatives are modified with an ether structure (carboxymethyl cellulose sodium, methylcellulose, hydroxypropyl cellulose, hydroxyethyl cellulose and ethyl cellulose) and esters structure (hydroxypropyl methylcellulose phthalate, cellulose acetate phthalate, cellulose acetate butyrate and cellulose acetate) [125,126]. The semi-synthetic polymers cellulose with ether of carboxymethyl cellulose sodium (CMC) has been studied with different kinds of hydrogen storage material for improving the hydrogen sorption performance [127]. The CMC is a non-toxic material, and it processes high viscosity properties. It is used as a binder to stabilize emulsions in numerous products because of its behavior/formation, such as thickener or viscosity modifier in desired conditions. CMC can probably be melted at a low temperature of nearly 547 K. Song et al. studied the impact of CMC on Mg-based hydrogen storage systems, where the hydrogen desorption and absorption behavior can be improved [128,129,130]. The combination of CMC and Mg has been prepared through a transformation involving milling. To understand the impact of CMC loading with Mg, two different kinds of compositions were made with CMC (95 wt % Mg + 5 wt % CMC-Mg-5CMC and 90 wt % Mg + 10 wt % CMC-Mg-10CMC) and without CMC (100 wt % Mg). As shown in Figure 3a, the Mg does not absorb any hydrogen at 523 K. However, the hydrogen absorption behavior of Mg has been altered under identical conditions after the inclusion of CMC. Compared to Mg-10CMC, the lower amount of CMC with Mg (Mg-5CMC) has been observed as more hydrogen in the initial period. By changing the hydrogen absorption temperature to 593 K, all hydrogen storage materials have significantly improved the hydrogen sorption kinetics (Figure 3b,c). The absorbed amount of hydrogen is 3.46, 7.38, and 5.25 wt % for 100 wt % Mg, Mg-5CMC and Mg-10CMC, respectively. CMC has enhanced the improvement of hydrogen sorption kinetics in Mg because of altering the Mg particle size significantly during the milling process [129]. To further increase the performance of the Mg-CMC composite, a desired amount of Ni has been added to the Mg-CMC composite. The addition of Ni in the Mg-CMC composite has enriched the hydrogen absorption and desorption kinetics [130,131,132].

## 4. Polystyrene

Setijadi et al. reported that Mg nanoparticles were stabilized by polystyrene for altering the thermodynamic properties [133]. The di-n-butylmagnesium is used as a source with polystyrene for Mg nanoparticles developments through the direct hydrogenolysis process. The Mg (particle size) formation has been effectively altered by the polystyrene during the decomposition of di-n-butylmagnesium with polystyrene in cyclohexane. The obtained particle sizes of Mg are approximately in the 25 to 50 range without polystyrene. The inclusion of polystyrene with an Mg source managed a larger-sized Mg particle formation of nearly 100 nm. The polystyrene modified the nucleation and growth process of Mg particle formation during the direct hydrogenolysis process, which led to 100 nm of Mg particle formation. Moreover, the as-prepared Mg–polystyrene was exposed to air to identify the impact of oxidation. The Mg nanoparticles were not degraded and showed stability after being exposed to air for 24 h. Interestingly, there was no significant evidence of MgO formation using XRD analysis. However, the XPS peaks revealed material oxidization, where the material can be oxidized reasonably. The polystyrene stabilized, and controllably oxidized Mg nanoparticles showed the considerably lowered enthalpy (52.3 ± 3.2 kJ mol^−1^ H_2_) and entropy (101.3 ± 4.5 J mol^−1^ K^−1^ H_2_), where the enthalpy and entropy of ball-milled MgH_2_ is 75.2 ± 1.8 kJ mol^−1^ H_2_ and 139 ± 3 J mol^−1^ K^−1^ H_2_, respectively [133,134]. The properties of polystyrene, mainly the absence of a highly reactive functional group, are the reason for the development of stabilized Mg nanoparticles, which provide stable hydrogen sorption performances and forbid the thermal degradation of Mg nanoparticles. More impressively, polystyrene protected the Mg nanoparticles from oxidation. The lower oxygen permeability properties in polystyrene (2.4 barrers) are the major factors for controlling the oxidation of Mg nanoparticles, but the polystyrene does not prohibit the hydrogen permeability [133,135]. Thus, polystyrene can be considered one of the efficient candidates to coat the hydride materials for controlling the reaction between hydride materials and oxygen.

## 5. Polyimide

The polyimide film has been used to prepare the Mg-based multilayer film with a different catalyst [136]. Polyimide possesses numerous advantages, such as high glass transition temperature, heat resistance, mechanical stability and chemical stability [137,138]. Hashimoto et al. developed the different kinds of Mg-based multilayer film with polyimide, namely polyimide-Mg, polyimide-Mg-Ti, polyimide-Mg-Pd, and polyimide-Mg-Ti-Pd through pulsed laser deposition [136]. This study used polyimide as a base material to coat the hydrogen storage materials and prepared the multilayer concepts. The binary catalysts of Ti and Pd have efficiently generated the synergistic effect to alter the hydrogen sorption kinetics. The Pd helps easier hydrogen absorption in Mg because of its reaction toward hydrogen molecules, where hydrogen molecules can effectively dissociate by Pd. The Ti catalyst enables the lower hydrogen desorption temperature of Mg. The polyimide film in Mg-based multilayer has not participated in the hydrogen storage behavior. However, it is an efficient base material for the multilayer film development. According to this concept, a sandwich type of hydride material concepts such as polymer film–hydride coating–polymer coating can be possibly established [136].

## 6. Polypyrrole

**Figure 4:**
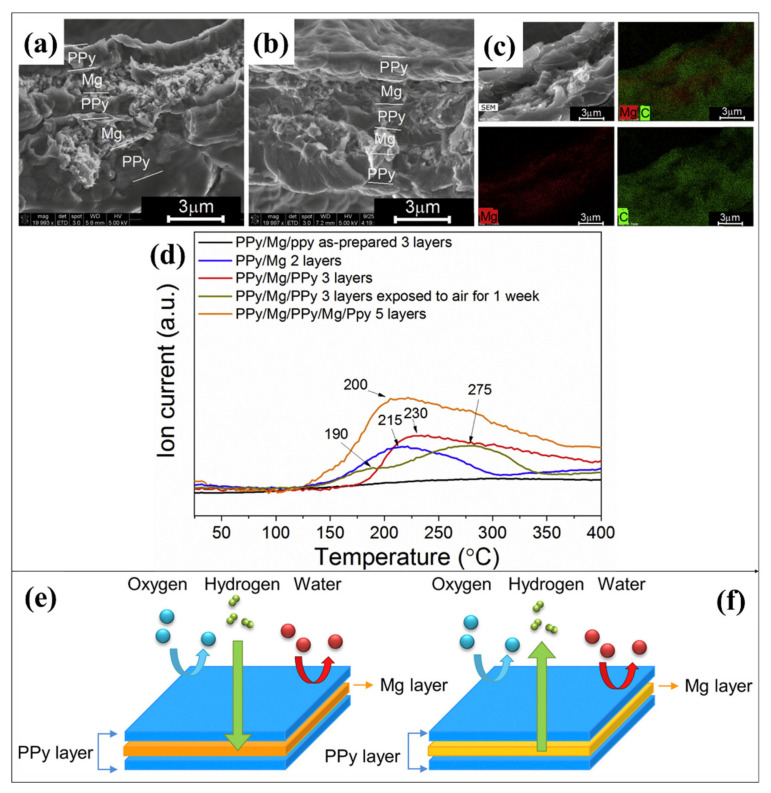
(**a**,**b**) SEM image of electrochemically developed PPy/Mg/PPy/Mg/PPy sandwich structure. (**c**) SEM cross-sectional mapping of PPy/Mg/PPy/Mg/PPy. (**d**) Hydrogen desorption of hydrogenated 3 and 5 layers PPy/Mg before and after air exposure. Schematic illustration of the impact of polymers during hydrogen (**e**) absorption and (**f**) desorption in Mg. Reprinted with permission from Ref. [139]. Copyright © 2018 Hydrogen Energy Publications LLC. Published by Elsevier Ltd. (License Number: 5305041063861).

A new concept of sandwich-type film of magnesium/polypyrrole (Mg-PPY) has been prepared with multilayered film formation through electrochemical deposition [139]. The Mg has stored in between the PPY to protect from oxygen and moisture. The Mg deposited on electropolymerized PPY films and the covering of PPY act as a protective polymeric layer for the Mg/MgH_2_. The multilayer Mg-PPY film was prepared through the electrochemical synthesis process. The titanium electrode has been used as a substrate to coat the electrically conductive polymer of PPY, and the nickel foil served as a counter electrode. The pyrrole monomer tetrabutylammonium hexafluorophosphate in tetrahydrofuran solution was used to deposit the PPY film on the Ti surface. The operating condition of 4 mA cm^−2^ for 2 min has been used for the electropolymerization of PPY. After the cleaning and drying process, the Mg grown on the PPY film surface and deposition of Mg has been carried out at 0.5 mA cm^−2^ using MgBu_2_ in THF as an electrolyte and a counter electrode of Mg ribbon. The as-prepared PPY layer was on Ti substrate, while the Mg layer was on PPY and the Mg layer was covered by PPY. Firstly, approximately 1 μm thickness of the PPY layer has been deposited in Ti substrate, as shown in the SEM micrograph images Figure 4a–b. Similarly, five layers of PPY and Mg have been developed, and the Mg layer is sandwiched between the PPY layers. The thickness of each PPY and Mg is nearly 1 μm. The SEM cross-sectional view and elemental mapping analysis confirmed the multilayer coating of PPY and Mg (Figure 4c). More impressively, magnesium’s hydrogen absorption and desorption temperature have been effectively altered by the PPY layer without the addition of any catalyst materials. The presence of the Mg layer sandwiched between PPY layers observed the hydrogen nearly at 100 °C. Moreover, the desorption temperature of Mg has decreased significantly, where the onset desorption starts at 125 °C and the maximum hydrogen release was observed at 215 °C (Figure 4d). The hydrogen sorption performance of Mg in the sandwich concept is considerably lower than the bulk Mg. The hydrogenated PPy/Mg/PPy film showed a stable nature after exposure to air for 7 days. The possible hydrogen sorption reaction has been schematically illustrated in Figure 4e,f. As shown in Figure 4e, the crossover of oxygen and water molecules through electropolymerized PPY film is negligible, effectively protecting the Mg/MgH_2_ from oxygen and moisture [139,140].

## 7. Polyvinylpyrrolidone

**Figure 5:**
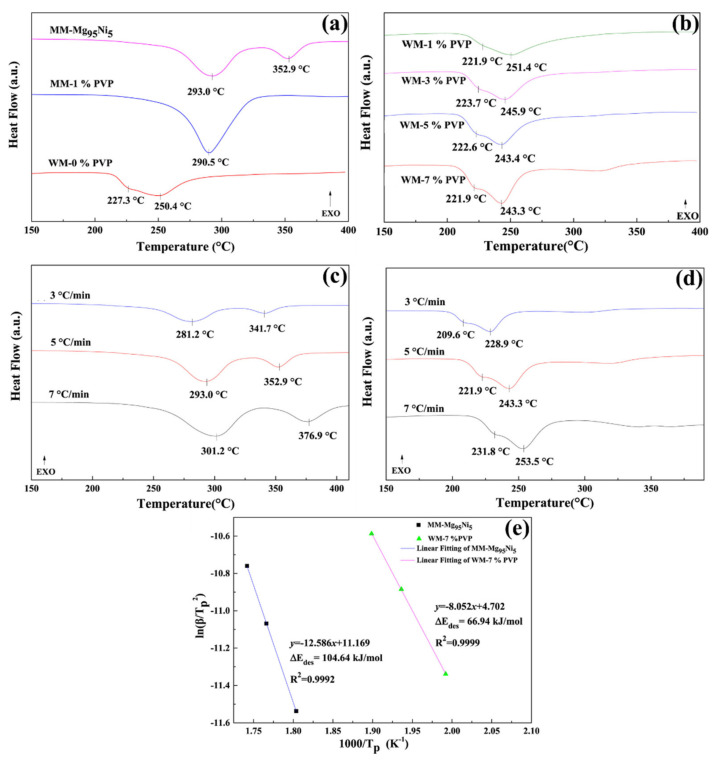
(**a**) DCS curves of mechanical milled (MM) and wet mechanical milled (WM) Mg_95_Ni_5_ with a heating rate of 5 °C (**b**) DSC curves of WM-Mg_95_Ni_5_ with the inclusion of different amount of PVP. Different heating rate DSC analysis of (**c**) MM-Mg_95_Ni_5_ and (**d**) WM-Mg_95_Ni_5_-7%PVP under similar conditions. (**e**) Kissinger’s plots of MM-Mg_95_Ni_5_ and WM-Mg_95_Ni_5_-7%PVP (dehydrogenation). Reprinted with permission from Ref. [141]. Copyright © 2018 Chinese Materials Research Society. Published by Elsevier B.V. (License Number: 5305060418416).

Yao et al. have reported the dehydrating behavior of Mg-Ni nanocomposite hydrogen storage material with polyvinylpyrrolidone (PVP) [141]. Two kinds of preparation processes have been utilized to prepare the hydrogen storage material and composite: hydriding combustion synthesis and wet ball milling process. Furthermore, the performance has been evaluated by changing the amount of PVP and the addition of tetrahydrofuran (THF) during the wet mechanical milling process. In the ball-milling process, with the inclusion of PVP with Mg_95_Ni_5_, a decrease in crystallite size has been observed in MgH_2_. The crystal sizes of MgH_2_ were determined as 23 nm and 18 nm without and with PVP, respectively. As shown in Figure 5a, the mechanically milled Mg_95_Ni_5_ revealed the maximum hydrogen desorption at 293 °C. Additionally, including PVP decreases the maximum desorption peak to lower temperatures. However, a significant change in desorption temperature has been realized in wet milling (WM) using THF. The maximum hydrogen description temperature peak was obtained at 293 and 250.4 °C at 5 °C/min for mechanical and wet milling hydride materials, respectively. In wet milling, the addition of PVP further decreases the dehydrogenation temperature. To evaluate the advantage of PVP, a different amount of PVP (1%, 3%, 5% and 7%) has been studied with Mg_95_Ni_5_ in the wet milling process. As compared to wet milling without PVP, the endothermic peak has been shifted to lower temperature at 251.4 °C, 245.9 °C, 243.4 °C, and 243.3 °C for 1%, 3%, 5% and 7% PVP, respectively, at 5 °C/min (Figure 5b). The hydrogen desorption temperature gradually decreases by increasing the PVP content in wet milling. The 7% PVP has been shown a better performance than the 1 and 3% PVP and similar performance to the 5% PVP. To further understand the impact of PVP addition, the activation energy has been calculated for mechanically milled Mg_95_Ni_5_ and wet milled Mg_95_Ni_5_ with THF and 7% PVP, as shown in Figure 5c,d. The activation energy for Mg_95_Ni_5_ and Mg_95_Ni_5_-THF-7% PVP is 104.64 and 66.94 kJ/mol, respectively (Figure 5e). This study proves the impact of THF and PVP on lowering the hydrogen desorption temperature, which helped to change the crystal size and particle sizes of Mg_95_Ni_5_. The THF and PVP produced a synergistic effect during the wet milling process, mainly enhancing dispersibility and prohibiting nanoparticles’ aggregation. Moreover, the PVP has a gas-selective barrier, effectively controlling the oxygen permeability [141]. This property in PVP can provide high air stability for MgH_2_ during exposure to air.

## 8. Polyvinylidene Fluoride

To improve the hydrogenation and dehydrogenation properties of Mg, the highly non-reactive thermoplastic fluoropolymer of polyvinylidene fluoride (PVDF) has been used as an additive in another approach. Song et al. reported the effect of PVDF with Mg by introducing 5 wt % of PVDF with 95 wt % Mg [142]. The composite has been developed through a reactive milling process in the hydrogen atmosphere. During the ball milling of Mg and PVDF, the polymer facilitated the cracks and defects in the hydride materials. The cracks and reduced particle size can generate a new and highly reactive surface, which shortens the path to an interaction between the hydrogen and Mg, effectively influencing the hydrogen absorption process. The formation of higher defects in the hydride materials can ease the nucleation. The presence of PVDF with Mg has effectively altered Mg’s hydrogen absorption and desorption performances, which may be attained by the change of surface structure and prevention of Mg aggregation by the PVDF. Thus, PVDF can also be considered one of the efficient polymer materials for developing the polymer–hydride composites for improving the hydrogen sorption performance in hydrides [143].

## 9. Polymethylpentene

**Figure 6:**
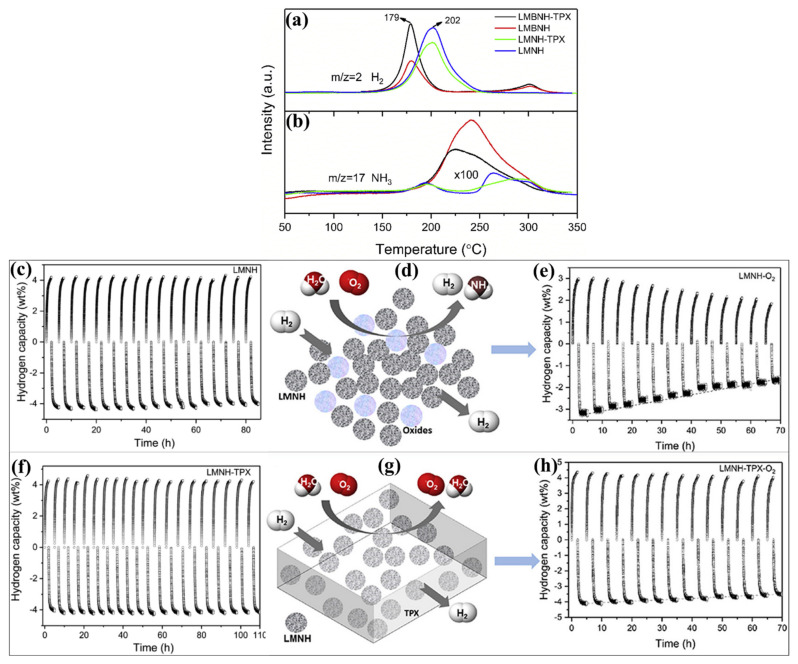
(**a**) H_2_-TPD-MS and (**b**) NH_3_-TPD-MS of LMNH, LMNH-TPX, LMBNH and LMBNH-TPX. Cyclic performers of LMNH without polymer: (**c**) as-prepared LMNH and (**e**) after exposure to air LMNH-O_2_. Cyclic performers of LMNH with TPX polymer: (**f**) as-prepared LMNH-TPX and (**h**) after exposure to air LMNH-TPX-O_2_. Schematic representation of polymers’ effect: LMNH (**d**) without and (**g**) with TPX polymer composition. Reprinted with permission from Ref. [143]. Copyright © 2018 Elsevier Ltd. (License Number: 5305110885251).

Polymethylpentene (the commonly used trademark name—TPX^TM^) has been considered an efficient polymer material and is widely used for various applications because of its favorable properties such as lightweight thermoplastic polymer, high melting point, thermal stability, good chemical resistance and transparency [144,145] (https://www.materialshub.com/material/polymethylpentene/, accessed on 1 May 2022). Polymethylpentene showed considerable benefits in the hydrogen storage system, which has been used as a host polymer [143]. Most likely, the polymethylpentene can protect the hydrides from moisture and oxygen. The Mg contained reactive hydride composite of Mg(NH_2_)_2_–LiH has been studied with polymethylpentene [143]. To identify the impact of reactive hydride composite, different compositions and amounts of the hydrides Mg(NH_2_)_2_–2LiH (LMNH) and 6Mg(NH_2_)_2_–9LiH–LiBH_4_ (LMBNH) have been chosen. These reactive hydride composites LMNH and LMBNH have efficient advantages such as working at lower temperatures (<250 °C), reasonable hydrogen capacity (gravimetric hydrogen amount nearly 5.6 wt %) and enthalpy of dehydrogenation (38.9 kJ/mol H_2_) [146]. The polymethylpentene with Mg(NH_2_)_2_–LiH revealed notable performances after exposing the composite to the air. Moreover, the kinetics and hydrogen capacity (related to the material weight) of reactive hydride composite is not affected by the inclusion of hydrides in polymethylpentene. Figure 6a,b represent the H_2_-TPD-MS and NH_3_-TPD-MS of LMNH and LMBNH. As shown in the figure, the onset and maximum decomposition peak of LMNH and LMBNH are more similar after including the polymer. The onset, maximum decomposition peak and end temperature peak of LMNH are 140, 202, and 250 °C, respectively, which is similar to the LMNH–polymer composite. The onset, maximum decomposition peak and end temperature peak of LMBNH are 130, 176 and 200 °C, respectively, which is similar to the LMBNH–polymer composite. To determine the reversibility measurements of LMNH and LMBNH with and with polymer, the performance of composite materials was determined before and after exposure to air. The cyclic sorption measurements were carried out at 20 °C temperature, and 1 and 80 bar pressure were used for desorption and absorption measurements, respectively. Figure 6c shows that the reactive hydride composites LMNH showed better cyclic performances. However, the cyclic performance has gradually deteriorated in the LMNH after exposure to the atmosphere (Figure 6e). In the case of LMNH–polymer, stable hydrogen sorption has been observed even after exposure to the air, as shown in Figure 6f,h. In this case, polymethylpentene polymer has protected the reactive hydride composites from the air and moisture (Figure 6g) [143]. It proves that the air-stable polymers can effectively control hydride contamination, which is a significant benefit for hydrogen storage systems.

## 10. Poly(Methyl Methacrylate)

**Figure 7:**
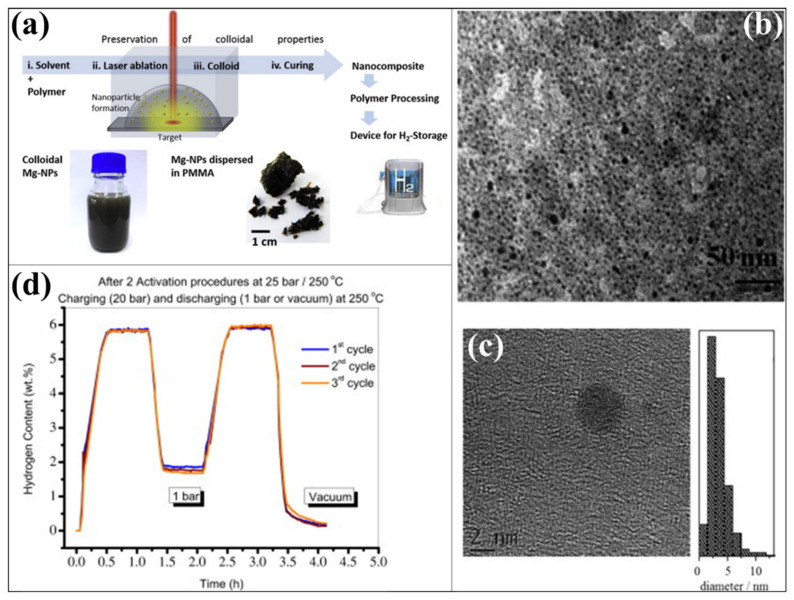
(**a**) Overall process of Mg-PMMA preparation through laser-based process. (**b**) TEM (**c**) HR-TEM and (**d**) hydrogen sorption (in magnetic suspension balance) characteristics of Mg-PMMA nanocomposite. Reprinted with permission from Ref. [154]. Copyright © 2013 Hydrogen Energy Publications, LLC. Published by Elsevier Ltd. (License Number: 5305160776309).

PMMA has been considered one of the efficient host, encapsulation or composite polymer materials for different kinds of solid-state hydrogen storage systems such as intermetallic hydrides, elemental hydrides, complex hydrides and chemical hydrides [124,147,148,149,150,151,152]. In the past two decades, the effectiveness of PMMA in Mg-based hydrogen storage has been proved by different approaches (such as the one-pot reduction process, laser ablation, mechanical milling and in situ solution reduction method), and it has shown numerous advantages because of its excellent functional properties [153,154,155]. PMMA is a kind of engineering plastic developed through the polymerization of methyl methacrylate. The synthetic polymer of PMMA is one of the transparent thermoplastic materials. It possesses numerous advantages such as lightweight (density of 1.17–1.20 g/cm^3^), excessive chemical stability, efficient mechanical stability (low elongation at break and greater Young’s modulus), non-toxicity and biocompatibility [156,157,158,159]. More impressively, the PMMA owns important characteristics of easy processing as a polymer moiety and compatibility, which are the most needed and advantageous behavior during polymer and inorganic materials composition [157]. In 2011, Jeon et al. established air-stable magnesium nanocomposites that include polymer materials to enhance the hydrogen storage capacity and reaction kinetics [153]. The authors identified the efficient performances of the magnesium composite without the high cost of heavy-metal catalysts. The Mg/PMMA nanocomposite was synthesized from an organometallic Mg^2+^ precursor and soluble organic polymer through a one-pot reduction process. For developing the Mg/PMMA nanocomposite, the precursor materials bis(cyclopentadienyl)magnesium (Cp_2_Mg) and PMMA were homogeneously included in the tetrahydrofuran solution. The lithium naphthalide was used as a reducing agent, and the reaction was carried out at room temperature. The nanocrystal of Mg has been developed in the solution containing PMMA by a burst nucleation and growth mechanism. The technique has prepared a mean diameter of 4.9 ± 2.1 nm of Mg nanocrystals on the PMMA. This progress has also encouraged a homogeneous dispersion of Mg nanocrystals throughout the polymer matrix without severe agglomeration. Compared to bulk Mg (44 μm) particles, the Mg/PMMA nanocomposite provided the most significant performance in the hydrogen absorption reaction under identical conditions. In the absorption condition of 200 °C and 35 bar, the Mg/PMMA nanocomposite absorbed over 3 wt % hydrogen for the first 5 min. The hydrogen absorption reaction has been continued for nearly 80 min, demonstrating ≈6 wt% of hydrogen uptake by Mg/PMMA nanocomposite, where the bulk Mg does not absorb hydrogen under similar conditions. More interestingly, a considerable amount of the magnesium oxide and magnesium hydroxide layer has not been detected after exposing the Mg/PMMA nanocomposite to air for specific periods. Here, the PMMA played multiple roles with Mg nanocrystals in the Mg/PMMA nanocomposite, such as the capping ligand, enabling the efficient hydrogen sorption kinetics without the high cost and heavy-metal catalysts, improving the hydrogen storage capacity and air-stable properties [153,160]. In another approach, Markridis et al. reported the Mg/PMMA nanocomposite via laser ablation [154]. To prepare the Mg nanoparticles into the PMMA, the polymer-containing solution was used, and the solution was positioned over the metal target. In this case, the top of the metal target is covered by the polymer-containing solution, as shown in Figure 7a. The metal target is focused by the pulsed laser beam, where the solvent is presented on the top of the target. The nanoparticle formation has been observed as follows: initially, the target materials absorb the laser pulse energy, and then, the vaporization of the target material occurs. Afterwards, the vaporized target materials condensed in the solvent contain the polymer materials. Upon this process, a desired size of the nanomaterials can be developed. As represented in Figure 7b, the Mg nanoparticles are homogeneously distributed in the overall PMMA matrix without a significant accumulation. Moreover, well-dispersed and uniform-sized Mg nanoparticles were exhibited. Interestingly, the size of the as-developed Mg nanoparticle in the Mg/PMMA nanocomposite is less than 5 nm (Figure 7c). The hydrogen sorption kinetics and uptake amount of Mg/PMMA nanocomposite by laser ablation showed considerable results, as shown in Figure 7d. The PMMA polymer encapsulated Mg nanoparticles showed considerable hydrogen uptake in less than 20 min at 250 °C and efficient hydrogen uptake content of approximately 6 wt % in Mg [154].

**Figure 8:**
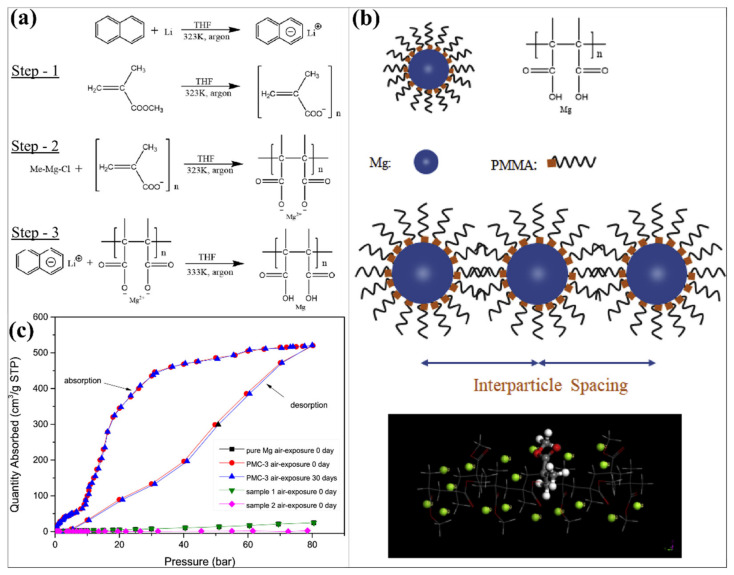
(**a**,**b**) Synthesis and formation process of PMMA-Mg NPs composite. (**c**) Hydrogen sorption of hydrides. Reprinted with permission from Ref. [162]. Copyright © 2019 Hydrogen Energy Publications LLC. Published by Elsevier Ltd. (License Number: 5305190661399).

Understanding the impact of different encapsulating polymers and the amount of Mg in the polymer matrix is detailed by Ruminski et al. [161]. Different polymers, namely, poly(methyl methacrylate), polyethylene (PE), polystyrene (PS) and polylactic acid (PLA), made composites with Mg and evaluated the effects. Interestingly, the mg-PMMA showed the efficient hydrogen uptake amount and kinetics compared to other polymers with Mg. The hydrogen uptake amount of Mg-PMMA (65% Mg), Mg-PE (62% Mg), Mg-PS (77% Mg) and Mg-PLA (65.1% Mg) is 6.95, 3.94, 5.63 and 0.58 wt %, respectively, under similar hydrogen sorption conditions. To considerate the loading quantity, a different amount of Mg (33.2 ± 0.9, 49 ± 1, 54.7 ± 0.6, 58.2 ± 0.6 and 65 ± 2 wt %) developed in the PMMA polymer matrix. By decreasing the PMMA amount in the Mg/PMMA nanocomposite, the hydrogen uptake amount is gradually increased because of the increment of Mg in the composite. It reveals the advantage of higher hydrogen uptake with polymer materials and reaching the theoretical limit. The hydrogen absorption amount of 33.2 wt % of Mg in Mg-PMMA and 65 wt % of Mg in Mg-PMMA is 4.86 wt % H_2_ and 6.65 wt % H_2_, respectively. Most importantly, the air stability has highly improved to the Mg by using a low amount of polymer encapsulated in the nanocomposite. The 65 wt % of Mg in Mg-PMMA showed highly air-stable behavior after exposure to air for 3 months, where the materials showed minor oxidation only. However, 33.2 wt % of Mg in Mg-PMMA showed poorer air-stable performances. The efficient performances (hydrogen uptake amount and steadiness) has been generated by arising the synergistic effect in 65 wt % of Mg in the Mg-PMMA composite because of enhanced interfacial structure templating areas and altering the paths of gas molecules (increase in the tortuosity) [161]. In another approach, a Mg-PMMA nanocomposite with a porous structure was developed through an in situ reduction process by reducing the Mg precursor (methyl magnesium chloride) to Mg nanoparticles, as shown in Figure 8 [162]. Li-naphthalene and Mg ion have been excellently mixed to prepare the efficient dispersion of Mg nanoparticles in the PMMA. In this case, approximately 5 nm of Mg nanoparticles developed in the PMMA. The Mg-PMMA nanocomposite formation mechanism has been schematically illustrated in Figure 8a,b. As shown in the figure, the PMMA dissolved in the solution and produced stable oxygen radicals. An electrostatic attraction occurred between the acrylate ions of MMA and organic Mg ions. Afterwards, the Mg nanoparticles are considerably enabled in the PMMA solution. Then, Mg coordinates with PMMA’s oxygen atom, which is the reason for arranging the molecular layer of PMMA on the Mg surface. Figure 8c represents the hydrogen sorption measurements of pure Mg and Mg-PMMA nanocomposite (before and after 30 days of air exposure) at 200 °C. The hydrogen sorption capacity and kinetic of 30 days of air exposure of the Mg-PMMA nanocomposite is closely similar to the Mg-PMMA nanocomposite (0-day air exposure) and pure Mg (0-day air exposure). This study proved that PMMA could effectively prohibit the interaction between the Mg and H_2_O/O_2_ and enhance the reaction kinetics during the hydrogen sorption process [162].

**Figure 9:**
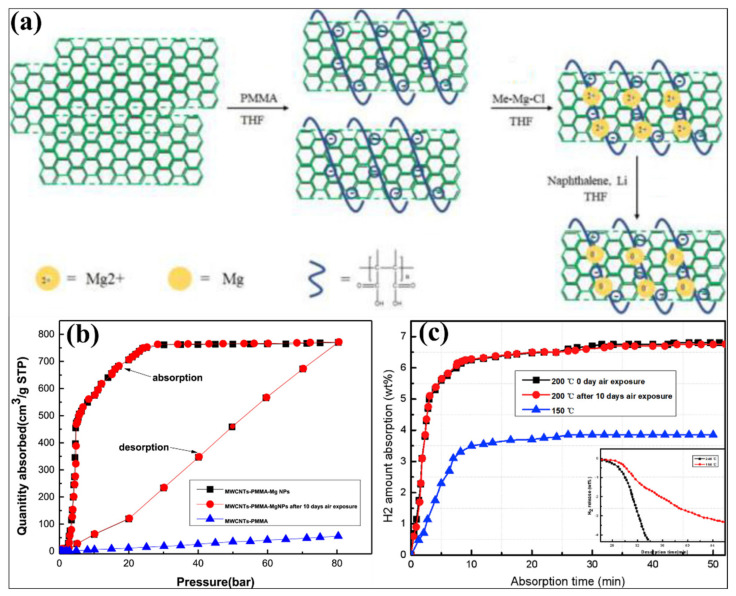
(**a**) Reaction process of MWCNTs-PMMA-Mg NPs composite synthesis. (**b**) PCI hydrogen sorption and (**c**) hydrogen absorption before and after 10 days of air exposure of MWCNTs-PMMA-Mg. Reprinted with permission from Ref. [163]. Copyright © 2019 Hydrogen Energy Publications LLC. Published by Elsevier Ltd. (License Number: 5305201060334).

**Figure 10:**
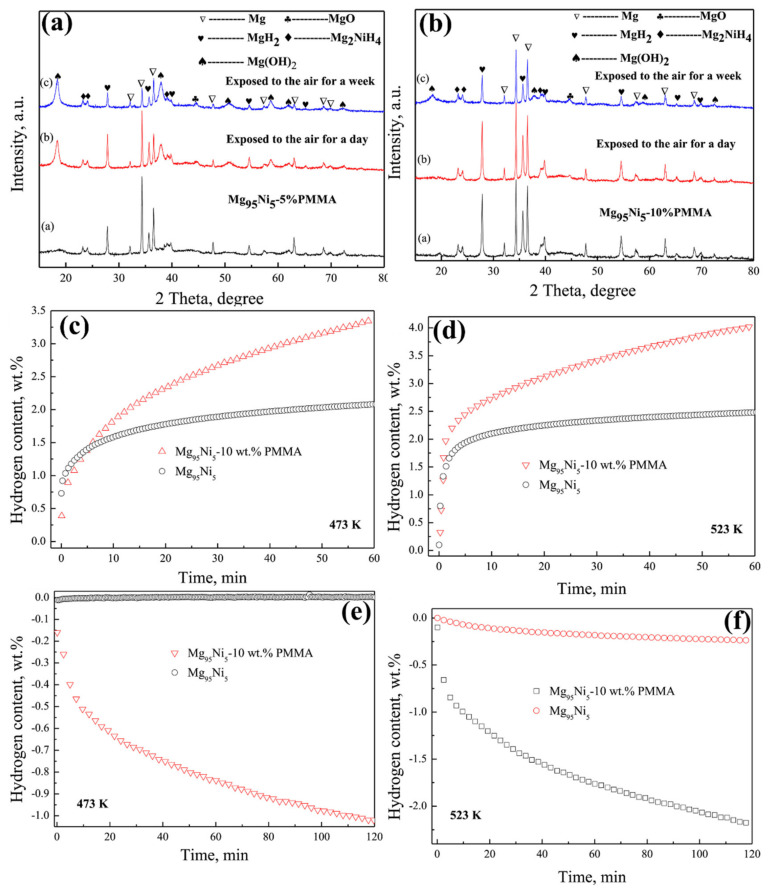
XRD patterns of before and after exposure to air: Mg_95_Ni_5_ with (**a**) 5 wt % PMMA and (**b**) 10% PMMA. The hydrogen absorption (**c**,**d**) and desorption (**e**,**f**) curves of Mg_95_Ni_5_ and Mg_95_Ni_5_- 10 wt % PMMA at different temperatures. Reprinted with permission from Ref. [165]. Copyright © 2017 Hydrogen Energy Publications LLC. Published by Elsevier Ltd. (License Number: 5305211052770).

Apart from the only Mg and PMMA nanocomposite, the developments of Mg with PMMA and multi-walled carbon nanotubes (MWCNTs) have also been explored [163]. Liang and co-workers developed the Mg nanoparticles from the methyl magnesium chloride on the porous MWCNTs-PMMA template, as shown in Figure 9a. This technique uses PMMA as an inexpensive surfactant for effectively dispersing the MWCNT and introducing the air-stable behavior for Mg hydrides. As reported, the observed size of the as-prepared Mg nanoparticles on the surface of MWCNTs-PMMA is about 3.8 nm, and it has been homogeneously distributed. The hydrogen sorption performance of Mg-MWCNTs-PMMA before and after 10 days of air exposure was evaluated at 200 °C from 0.01 bar to 80 bar pressure, as shown in Figure 9b. The as-prepared material (for Mg NPs in Mg-MWCNTs-PMMA) showed a hydrogen uptake of 7.1 wt % (750 cm^3^/g STP). The Mg-MWCNTs-PMMA exposed in air for 10 days has also shown similar hydrogen sorption performances under similar conditions. In addition, to confirm the impact of the polymer, the hydrogen absorption performance has been studied for the non-air exposed and air-exposed Mg-MWCNTs-PMMA materials (Figure 9c). In this case, the Mg-MWCNTs-PMMA (0-day air exposure) revealed higher hydrogen uptake of 6.7 wt %, which was excellent hydrogen uptake at 200 °C. Remarkably, similar hydrogen absorption amount and kinetics have been observed to the Mg-MWCNTs-PMMA (after 10 days of air exposure) under similar conditions. It has been proved that the presence of MWCNTs and PMMA creates a synergistic effect for enhancing the hydrogen sorption kinetics, improving the quantity of hydrogen uptake amount and prohibiting the oxidation of Mg during the exposure to air [163]. In another report, the impact of Ni additive (different amount) in the Mg-PMMA composite and the effect of varying the ball milling time was studied by Raygan and Azar [155]. The confinement of Mg nanoparticles in PMMA polymer effectively influences hydrogen sorption behavior. PMMA is a barrier for forming Mg oxide or hydroxide during atmospheric air exposure. Moreover, adding Ni in the Mg-PMMA nanocomposite influences the hydrogen sorption kinetics [155]. For a different (electrochemical properties) application, an alloy of Mg_3_MnNi_2_ is coated by a different ratio of PMMA-MWCNTs, where the obtained coating thickness is 30 nm on the surface of the Mg_3_MnNi_2_ [164]. These kinds of concepts can also be considered for the hydrogen storage application. As reported by Yuan et al., the Mg-based hydride was developed by mechanical milling of the hydriding combustion synthesized (HCS) process [165]. In this case, approximately 100 nm of Mg_95_Ni_5_ nanoparticles have been developed. The particle size and distribution of Mg_95_Ni_5_ have varied by varying the PMMA amount, where the particle size and efficient distribution were obtained using a higher content of PMMA. Figure 10a,b represents the XRD images of different wt % of PMMA (5% and 10%) with Mg_95_Ni_5_ before and after exposure to air. The Mg_95_Ni_5_-10 wt % PMMA showed the considerable stability than the Mg_95_Ni_5_-5 wt % PMMA after exposure to air for a day. In Mg_95_Ni_5_ with 5 wt % PMMA, the MgO and Mg(OH)_2_ peaks have been highly observed after being exposed to air. On the other hand, a very smaller amount of intensity of MgO and Mg(OH)_2_ peaks were detected for 10 wt % PMMA-Mg_95_Ni_5_ after exposure to air. It has been proved that the 10 wt % PMMA provides an efficient antioxidant and water resistance capability. Comparatively, the hydrogen absorption kinetics and hydrogen uptake amount of 10 wt % PMMA-Mg_95_Ni_5_ is significantly higher than the Mg_95_Ni_5_ under identical conditions, as shown in Figure 10c,d. The hydrogen uptake amount of Mg_95_Ni_5_ and 10 wt % PMMA-Mg_95_Ni_5_ is 2.48 wt % (2.08 wt %) and 4.04 wt % (3.37 wt %), respectively, at 523K (473K) for 60 min under identical conditions. Similar to the hydrogen absorption kinetics, an excellent hydrogen desorption kinetics was obtained at 10 wt % PMMA-Mg_95_Ni_5_ compared with the Mg_95_N_i5_ (Figure 10e,f). The reaction kinetics and improved hydrogen uptake/release content in 10 wt % PMMA-Mg_95_Ni_5_ are mainly attributed to preventing the aggregation of hydride nanoparticles by PMMA polymer materials and the uniform dispersion of hydride material in the polymer, which improves the nanostructure effects [165]. The hydrogen sorption information of the PMMA polymer with Mg-based hydrogen storage materials has been represented in Table 2. Typically, the polymers (such as CMC, polystyrene, polyimide, PPY, PVP, PVDF, TPX^TM^ and PMMA) provide significant advantages for Mg-based hydrogen storage systems in various factors. Apart from the above polymer materials, multiple studies need to be conducted for Mg-based hydrides using different kinds of polymers. The polymers—namely, acrylonitrile butadiene styrene copolymer (ABS), acrylonitrile-EPDM (ethylene/propylene/diene)-styrene (AES), polytetrafluoroethylene (PTFE), polyacetylene (PA), poly(*p*-phenylene) (PPP), poly(diphenylacetylene) (PDPA), polyaniline (PANI), polyethylene (PE), polyethylenimine (PI), polyetherimide (PEIS), polyacrylonitrile, poly(acrylonitrile-*co*-butadiene-*co*-acrylic acid) (PABA), poly (methyl methacrylate)–*co*–butyl methacrylate) (PMMA–co–BM), ethyl cellulose and elastomer can also be considered for the Mg-based hydride system, where these polymers have already proved their influence in other hydride systems [39]. The selection of polymer material needs a low-cost, abundant, easier surface modification, facile synthetic process, and higher thermal stability. Suppose the new kind of polymer possesses gas-selective properties and hydrogen sorption behavior. In that case, that will be a good choice for developing a new kind of polymer–hydride hydrogen storage materials where it can prevent the hydrides from contaminating and enhance the hydrogen storage capacity by avoiding the unreacted regions, respectively. Further developments and the selection of excellent polymers for hydrides need to be vigorously studied to successfully commercialize the different kinds of hydrogen storage materials.

## 11. Conclusions

The Mg-based materials/hydrides are proved to be an efficient candidate for hydrogen storage materials according to their realistic gravimetric and volumetric hydrogen density. Specifically, the Mg/MgH_2_ showed considerable attention in the hydrogen energy sectors because of its lightweight, high gravimetric hydrogen capacity (7.6 wt%), high volumetric hydrogen capacity (110 hydrogen/liter), and reversibility. However, high sorption temperature, sluggish sorption kinetics and contamination (during exposure to air) of Mg/MgH_2_ significantly affect its performance. Numerous techniques have been introduced to overcome the barriers in Mg-based hydrides for establishing practical applications. However, most of the efforts have been dedicated to altering the thermodynamic and kinetics-related issues of Mg-based hydrides. In recent periods, particular efforts have been made to overcome the contamination of Mg-based hydride during exposure to air using gas-selective polymers. This review article provides comprehensive information and the impact of various polymers on Mg-based hydrides. As per our intellectual awareness and literature collections, the polymers, namely, carboxymethyl cellulose, polystyrene, polyimide, polypyrrole, polyvinylpyrrolidone, polyvinylidene fluoride, polymethylpentene, and poly(methyl methacrylate) have been studied for Mg-based hydride systems. The polymer materials played a significant role in preventing the Mg-based hydrides from the contaminants such as oxygen, protic species and CO_x_. The Mg-based hydride–polymer system encapsulates the Mg-based materials by gas-selective polymers, where the Mg-based hydrogen storage materials are present inside the polymer matrix. In this case, the polymer materials allow only hydrogen gas in the polymer matrix to react with Mg-based hydrogen storage materials. The gas-selective polymers control the contaminants’ permeation (oxygen, protic species and CO_x_) and prohibit the reaction between Mg-based hydrogen storage materials and oxygen/protic species/CO_x_. In addition, the gas-selective polymer materials provide additional benefits in some cases of the Mg-based hydrides, mainly improving the sorption kinetics. Based on the advantages of this behavior in polymers, the hydrogen storage materials in the gas-selective polymer can be considered an excellent candidate for hydrogen storage materials for practical application. This comprehensive review provides excellent information and more understanding of the impact of polymers on Mg-based hydrogen storage materials.

## Figures and Tables

**Table 1:** US-DOE Technical Targets of Onboard Hydrogen Storage for Light-Duty Fuel Cell Vehicles [5] (US-DOE—Hydrogen and Fuel Cell Technologies Office).

Target	Year		
	2020	2025	Ultimate
Gravimetric capacity (wt %)	4.5%	5.5%	6.5%
Volumetric capacity (g/L)	30	40	50
Cost ($/kg H_2_)	333	300	266
Durability/Operability:			
• Operating temperature (°C)	−40/60	−40/60	−40/60
• Min/max delivery temperature (°C)	−40/85	−40/85	−40/85
• Operational cycles	1500	1500	1500
• Min/max delivery pressure (bar)	5/12	5/12	5/12
• Onboard efficiency	90%	90%	90%
Charging/Discharging rate:			
• System fill time (min)	3–5	3–5	3–5
• Min full flow rate ((g/s)/kW)	0.02	0.02	0.02
• Average flow rate ((g/s)/kW)	0.004	0.004	0.004
• Start time to full flow @ 20 °C (s)	5	5	5
• Start time to full flow @ −20 °C (s)	15	15	15
• Transient response at operating temperature 10–90% and 90–0% (based on full flow rate) (s)	0.75	0.75	0.75

**Table 2:** Hydrogen sorption information of composites of Mg-based hydrides with PMMA polymer.

Hydrogen Storage Materials/Hydrides with PMMA Polymer	Hydrogen Sorption Information	Ref.
Mg NCs/PMMA composites	H_2_ absorption at 200 °C and 35 bar H_2_ absorption capacity value of 5.97 wt % Mg (≈4 wt % total)	[153]
Laser ablated nano Mg/PMMA composite	≈96% of the absorbed H_2_ amount was desorbed at 250 °C in less than 20 min	[154]
Mg–PMMA nanocomposites	6.95 wt % H_2_ absorption (200 °C, 30 bar H_2_)	[161]
Mg–polyethylene nanocomposites	3.94 wt % H_2_ absorption (200 °C, 30 bar H_2_)	
Mg–polystyrene nanocomposites	5.63 wt % H_2_ absorption (200 °C, 30 bar H_2_)	
Mg–polylactic acid nanocomposite	0.58 wt % H_2_ absorption (200 °C, 30 bar H_2_)	
Mg_95_Ni_5_-PMMA	Requiring 60 min to absorb 3.37 wt % H_2_ at 473 K Desorbing 1.02 wt % H_2_ within 120 min at 473 K	[165]
MWCNTs–PMMA–Mg NPs composites	H_2_ absorption capacity of 6.7 wt% at 20 bar and 200 °C Release 3.7 wt % of H_2_ at 150 °C and 0 bar H_2_	[163]
Mg NPs in porous PMMA	H_2_ storage capacity of about 4.8 wt % within 30 min Very rapid absorption kinetic (>4.0 wt %) within 8 min at 30 bar and 200 °C	[162]
MgH_2_–PMMA nanostructured composite	H_2_ desorption of over 6 wt % after heating up to 300 °C and holding for 15 min	[155]

## Data Availability

Not applicable.
